# Effects of mild therapeutic hypothermia on acute kidney injury after cardiopulmonary resuscitation

**DOI:** 10.1186/2197-425X-3-S1-A467

**Published:** 2015-10-01

**Authors:** J Hasslacher, F Barbieri, U Harler, G Lehner, S Dunzendorfer, M Joannidis

**Affiliations:** Medical University of Innsbruck, Joint Institution for Emergency Medicine and Critical Care Medicine, Innsbruck, Austria

## Introduction

About the effect of mild therapeutic hypothermia on renal function after cardiopulmonary resuscitation little data exists. From animal and human studies it is known that renal function might be impaired, which is not reflected by serum creatinine levels if creatinine production is reduced.

## Objectives

The aim of the study was to investigate the possible influence of mild therapeutic hypothermia (MTH) on the incidence of acute kidney injury (AKI) and renal function parameters [[1]].

## Methods

This is a retrospective analysis of prospectively collected data on patients after successful cardiopulmonary resuscitation (CPR) at our ICU. Serum creatinine and cystatin C were measured at baseline, daily up to 5 days and at ICU discharge. AKI was defined by the KDIGO criteria (only based on creatinine values). MTH was applied for 24 hours targeting a temperature of 33°C using an intravascular cooling device. Neurological outcome was assessed with the Cerebral Performance Categories (CPC) score at hospital discharge.

## Results

55 of 126 patients were treated with MTH, 20 patients received continuous renal replacement therapy, 62 of 126 patients had good neurological outcome (CPC 1-2). In patients treated with MTH there was a significantly lower incidence of AKI (9 vs 20; p = 0.007) accompanied by significantly lower creatinine levels on day 1-2 and at ICU discharge (day 1: 1.12 (0.90-1.29) vs. 1.29 (1.00-1.52) mg/dl; p= 0.016) and lower cystatin C levels on day 1-4 and ICU discharge (day 1: 0.88 (0.77-1.10) vs. 1.29 (1.06-2.16)mg/l; p < 0.001). Restricting analysis to patients with good neurological outcome confirmed a significant lower creatinine on day 2 (0.82 (0.67-1.02) vs. 1.03 (0.83-1.38) mg/dl; p = 0.049) and cystatin C on day 1-2 when treated with MTH. In patients with poor neurological outcome treated with MTH cystatin C levels were also significantly lower on day 1-2 (day 1: 0.96 (0.83-1.30) vs. 1.44 (1.17- 2.29) mg/l; p = 0.01) and at ICU discharge, whereas creatinine was only significantly lower at ICU discharge (0.84 (0.51-1.36) vs. 1.23 (0.87-2.15) mg/dl; p = 0.039). AKI occurred significantly less often (10 vs. 35; p = 0.017) in this subgroup.

## Conclusions

MTH seems to have a protective effect against the development of AKI, especially in patients with poor neurological outcome. Cystatin C seems to play a role for determination of renal function in patients treated with MTH.

**Figure 1:**
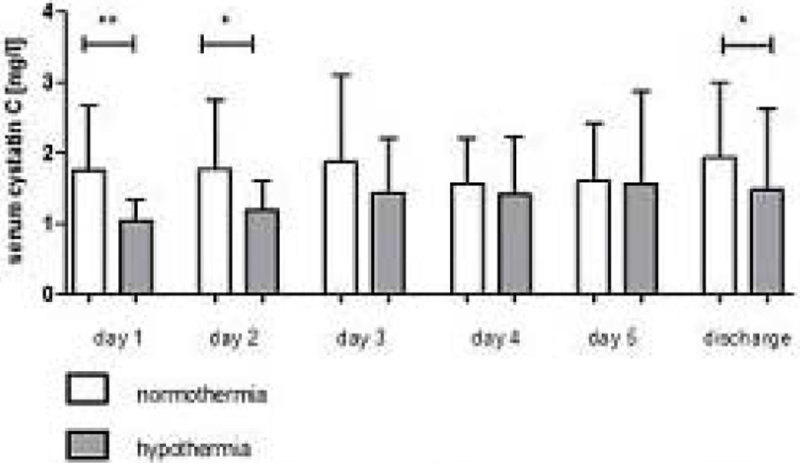
**Serum cystatin C levels (mg/l) on day 1-5 and ICU discharge in patients treated with mild therapeutic hypothermia or normothermia.**
